# Social Media Use and Monitoring for Adolescents With Depression and Implications for the COVID-19 Pandemic: Qualitative Study of Parent and Child Perspectives

**DOI:** 10.2196/21644

**Published:** 2020-12-08

**Authors:** Candice Biernesser, Gerald Montano, Elizabeth Miller, Ana Radovic

**Affiliations:** 1 Department of Psychiatry University of Pittsburgh Pittsburgh, PA United States; 2 Department of Pediatrics Division of Adolescent and Young Adult Medicine University of Pittsburgh Pittsburgh, PA United States; 3 Center for Behavioral Health, Media, and Technology University of Pittsburgh Pittsburgh, PA United States

**Keywords:** social media, adolescent, parents, depression, disclosure, qualitative research, COVID-19

## Abstract

**Background:**

Although youth report many positive experiences with social media (SM) use in their daily lives, adolescents with depression are more vulnerable to the risks of SM use than adolescents without depression. Parents protect adolescents with depression from the risks of SM use by monitoring their child’s SM activity; however, this comes into conflict with the adolescent’s need for autonomy in their web-based communication. The implications of SM use and monitoring for adolescents with depression and their parents are of particular relevance to the COVID-19 pandemic, as rates of SM use have increased in response to physical distancing measures.

**Objective:**

This study aims to explore parent and child perspectives regarding the use and function of SM in the daily lives of adolescents with depression and parents’ perceptions of and experience with monitoring their child’s SM use.

**Methods:**

We conducted qualitative interviews with adolescents with depression (n=23) and one parent of each adolescent (n=23) between July 2013 and September 2014. The adolescents were patients seeking treatment for depression in Pittsburgh, Pennsylvania. Data analysis included dyadic analysis of the adolescents’ and parents’ perspectives and qualitative descriptions of individual parent interviews to explore their experiences with SM use and monitoring. The construct of parental knowledge and factors hypothesized to contribute to parental knowledge, including adolescent disclosure, parental solicitation, and parental control, were used to guide the codebook and dyadic data analysis.

**Results:**

Dyadic analyses showed that parents and their children disagreed on the use and function of SM in the daily lives of adolescents with depression, with adolescents viewing SM as a forum for honest expression of their emotions, whereas parents felt that their children’s posts were inconsequential and interfered with the adolescents’ lives. Furthermore, parents reported using a wide range of strategies to gain knowledge of their child’s SM use to monitor their safety on SM, including direct solicitation and indirect solicitation, such as keeping the child's passwords, asking friends or siblings about their child's SM use, and restricting SM behavior and access to devices.

**Conclusions:**

Clinicians should support adolescents with depression and their parents in finding common ground for an effective and acceptable monitoring approach. Resources are provided for clinicians navigating conversations about SM use and monitoring with adolescents with depression and their parents during the COVID-19 pandemic.

## Introduction

### Background

Adolescents are widely reported to be ubiquitous users of social media (SM). As of 2018, 70% of US adolescents reported using SM multiple times per day, a rate that has doubled from reports in 2012 (34%) [1]. More than half of parents are concerned that their children spend too much time on their mobile phones and worry about the potential negative effects of screen time in their children’ s daily lives [2]. Parents of adolescents with depression may have unique concerns regarding their children’s ongoing consumption of SM, as they perceive heightened susceptibility to harmful experiences [3].

### Adolescent SM Use During the COVID-19 Outbreak

The use of SM during the COVID-19 pandemic is estimated to have increased substantially. In their analysis of SM use from web-based data providers, *The* *New York Times* reported that the use of the popular SM sites Facebook and YouTube increased by 27% and 15%, respectively [4]. Increasing rates of SM use may be especially meaningful to young people, as physical distancing measures limit face-to-face interaction. Deprivation of peer interactions, in particular, is challenging during adolescence, a developmental stage during which peer influence and acceptance are important [5]. In the face of these concerns, SM may play an influential role. Studies of adolescent SM use show that aspects of prosocial face-to-face interactions that are protective to adolescent mental health, such as social support, social reward, and reduction of feelings of social exclusion, can be mirrored through active engagement on SM [5]. Although SM may play an important role in mitigating limitations to social interaction during the pandemic, there are also concerns associated with higher rates of SM activity. Prevention groups have raised concern that stress associated with the pandemic could contribute to higher rates of negative SM experiences, such as cyberbullying [6]. The potential for harmful SM interactions is complicated by reduced access to counseling and mentorship from educators [6]. In the current context, considering adolescents’ SM use and the ways in which parents monitor their use is significantly important.

### Use of SM in Adolescents With Depression

Adolescents with depression face vulnerabilities that place them at a higher risk of having negative SM experiences and unique opportunities for social support and connection than youth who are not depressed. Adolescents with depression use SM in positive ways; for example, engaging in entertainment, humor, or content generation can act as a distraction from depressed mood [7], and these adolescents can also reach out to others for support in coping with depression or for social connection [8]. With regard to vulnerability to negative SM experiences, adolescents with depression are more likely to engage in risky SM behavior, such as talking to strangers and disclosing personal information [9], and to be exposed to potentially harmful SM content, such as self-harming behaviors [10,11]. In addition, youth with depression are more likely to report internet harassment [12], cyberbullying [13,14], and addictive internet use [15] experiences that have been associated with fluctuations in mood states and heightened suicidal risk among adolescents [16-20]. Parents of adolescents in treatment for depression or suicidal risk have reported awareness of their child’s heightened vulnerability to negative SM experiences and describe an urge to stay informed about their child’s SM use to protect them from harm [3].

### Parent and Adolescent Perceptions Toward SM Use and Monitoring

Adolescents and parents often exhibit different perspectives regarding SM use [2]. Adolescents tend to view SM in a more positive light, citing benefits of social connection, social support, and opportunities to express themselves [21]. In contrast, parents report more negative views toward SM considering its potential detrimental effects on youth [21]. The shifting parent-adolescent relationship that is typical of adolescent development may also contribute to these differences in perspectives on SM use. Adolescents seek freedom of expression on SM, whereas parents balance their child’s desire for independence with their own desire to protect their child from risks on SM [22]. Discrepancies in these views are likely to be unique when considering adolescents with depression and their parents. For example, adolescents with depression may value support they receive through SM and autonomy in privately engaging with trusted peers. Parents of adolescents with depression may be more attuned to potential negative aspects of SM use on their child’s depression and may also perceive a strong need to monitor.

### Parental Monitoring and Knowledge of Adolescent’s SM Use

Parental monitoring has been defined as the tracking or surveillance of a child’s behavior and is often assessed through measures of parental knowledge [23]. Kerr and Stattin [23,24] hypothesize 3 primary sources of parental knowledge. These sources include (1) adolescents’ voluntary sharing of information with their parents; (2) parents’ solicitation of information from their child; and (3) parents’ use of control, meaning setting rules and restrictions to limit their child’s ability to engage in activities without informing their parents [23,24]. Thus, information sharing between parents and adolescents can occur through dialogue (eg, informal or formal monitoring discussions) or through behaviors (eg, parents’ engagement in mediation of SM as a means to gain control). Of these sources, parental knowledge is most strikingly informed by adolescent disclosure.

Voluntary adolescent disclosure is considered to be a strong and consistent predictor of parental knowledge above and beyond parents’ solicitation of information [25]. By the time of adolescence, youth are developmentally capable of making choices to voluntarily offer private information [26]. Keeping secrets, particularly when negative consequences are perceived, is also developmentally typical and impacted by contextual factors within the family, such as the degree of trust present between parents and adolescents and overall relationship quality [26,27]. Supportive parenting styles exhibited by warmth, affection, and responsiveness to adolescents’ needs are more likely to facilitate voluntary disclosure [28,29]. Unsupportive styles of parenting, such as parenting that involves the use of psychological control or manipulation techniques such as love withdrawal, shaming, or guilt induction, act as barriers to voluntary disclosure [28,29].

Some methods of parental solicitation and control have been associated with reductions in parents’ knowledge of their child’s SM behavior. Parents’ engagement in privacy invasions or the covert solicitation of a child’s private information often act as impediments to open parent-child communication. Privacy invasions have been associated with increased adolescent secrecy and deficits in family functioning, for example, problematic communication, behavior, and relationships [30-32]. Engaging in methods of parental control that adolescents may see as a restriction to their freedom of expression also hinders adolescent disclosure and, in turn, increases secrecy [33].

These long-standing trends toward parental knowledge and control remain consistent within media studies. In their meta-analysis, Chen and Shi [34] showed that parents’ active engagement with their child’s media use, namely, through openly discussing media use or parental co-use of media with their child, was effective in reducing SM risk behaviors. They also found that parental restriction alone was not associated with a reduction in risky behaviors.

#### Context of Parental Knowledge of SM Use Among Adolescents With Depression

Parental knowledge of SM use is of critical importance for the discussion of depression in adolescents. Open parent-child communication that is fostered through adolescents’ voluntary disclosure is associated with reductions in depressed mood, improvements in self-esteem, and positive and realistic expectations toward media use [23,24]. Conversely, parents’ engagement in unsupportive communication or deployment of control strategies (eg, setting rules that require their child to share information about their SM use) has been linked to increased depressive symptoms and poorer adjustment among adolescents [23,24,35]. When parents’ lack of support takes the form of negative reactions to adolescents’ disclosures, adolescents are left feeling disconnected to their parent and respond by engaging in secrecy [33]. Secrecy could be detrimental in situations when a parent may need to intervene to address their child’s depressed mood or suicidal thoughts.

### Research Gaps

Parents’ attempts to understand and explore their child’s SM use may be especially challenging in the current era in which SM activity is ubiquitous and trends toward use among adolescents are increasing in the face of the COVID-19 pandemic. Little is known, however, about the context of parental monitoring of SM use among adolescents with depression. Understanding the context of how parents of adolescents with depression balance the unique challenges in weighing their child’s unique vulnerabilities toward negative SM experiences and unique opportunities for social support and connection could offer important contributions for clinicians who are partnering with families to support youth with depression. First, similarities and differences in perspectives of adolescents with depression and their parents toward SM use and monitoring have not been explored. Considerations toward monitoring among youth with depression, who are more vulnerable to negative SM experiences, may be distinct from adolescents who are not depressed. Second, strategies that parents of adolescents with depression use to stay informed of their child’s SM use are not well understood and may offer helpful guidance to parents of adolescents with depression on how to protect their child while supporting their emerging independence.

The exploration of these findings may be especially beneficial to providers engaged in the mental health care of adolescents who are recommended to assess the harmful and helpful influences of adolescents’ digital lives [36]. Providers’ ability to accurately assess the SM context is likely to be influenced by parent and child perceptions toward the use and function of SM. Furthermore, providers who wish to partner with parents to observe and protect adolescents’ SM environments may face challenges if the child’s and parent’s preferences toward SM monitoring strategies are discrepant.

### Aims

To offer insights for clinical practice with adolescents with depression and their families, we sought to explore the perspectives of youth with depression and their parents toward SM use and monitoring. Through interviews with adolescent and parent dyads, we aim to compare parent and child views toward the use and function of SM use in the daily lives of adolescents with depression. Through interviews with individual informants, we aim to explore parents’ strategies and experiences with gaining knowledge of their child’s SM use.

## Methods

### Recruitment and Sampling

As part of a larger qualitative study to inform an intervention using SM for adolescents with depression and their parents [7], a convenience sample of adolescents aged 13 to 20 years diagnosed with depression and currently receiving treatment in Pittsburgh, Pennsylvania, and one parent of each adolescent were invited to participate in interviews. From July 2013 to September 2014, potential participants were informed about the study by clinicians treating patients for depression at 2 sites: (1) an academic adolescent and young adult medicine clinic with mental health services available or (2) a specialty psychiatric clinic for adolescents with depression and suicidality. Of the 31 adolescent-parent pairs (of which there were 31 adolescents and 30 parents because 1 mother had 2 children in the study) who filled out an interest form, 8 adolescents and 7 parents could not be reached for an interview; thus, 23 adolescents and 23 parents completed the study. Of these, there were 21 adolescent-parent matched pairs. One parent had 2 children in the study. Reporting of data collection and analytic methods follows the Consolidated Criteria for Reporting Qualitative Research [37].

### Data Collection

All interviews were conducted by the senior author, who was trained and experienced in qualitative data collection and analysis. She introduced herself to adolescents as a researcher and physician in Adolescent Medicine. Semistructured interviews were conducted over telephone (n=17 adolescents and n=17 parents) or in person in a private patient room in the clinical setting (n=6 adolescents and n=6 parents). Parent and child interviews were conducted separately. We obtained verbal consent from parents for themselves and from adolescents aged 18 years and older; for those aged 18 years and younger, we obtained parental permission and adolescent assent. The interviewer assured participants that the research team would guard confidentiality, specifically not sharing phrases said on SM, which could potentially be searched to identify them.

A 30- to 60-minute semistructured interview, facilitated through an interview guide while remaining open to topical trajectories that broadened the understanding of participants’ perspectives, was conducted individually with the adolescent alone and then with the parent alone. These interviews were used to obtain information about SM use characteristics and positive and negative experiences with SM use for both adolescents and parents—pertaining to parents’ personal use of SM and whether the parent uses SM or the internet to learn more about their child’s depression or to connect with other parents. Parents were also asked (1) whether their child uses SM and to describe their positive and negative SM experiences, (2) their opinion of and experiences with their child sharing personal thoughts with others via SM, and (3) their experience communicating with their child through SM. Adolescents were asked about how the type and extent of their SM use varies with their mood. Both adolescents and parents were also asked to provide demographic information, including age, gender and race, length of depression treatment, mobile phone ownership, and primary device for internet use. Questions on SM use characteristics were adopted from Pew Research Center studies on adolescent SM behavior [38,39]. As compensation for study participation, adolescents received a book about adolescent with depression or bipolar disorder [40], and parents received a book about being a parent of an adolescent with depression or a bipolar disorder [41]. The study protocol was approved by the University of Pittsburgh Human Research Protection Office.

### Data Analysis

We analyzed interviews of both individual informants and dyads to explore adolescent and parent perspectives toward SM use and monitoring. Such an approach offers the potential to investigate rich contextual factors associated with concordant and discrepant family experiences [42]. Individual interviews are well suited for offering a contextual analysis that sheds light on perspectives from a specified group [43]. Exploration among dyads can enrich findings from individual interviews by offering an ability to compare across multiple perspectives, which is known to improve the trustworthiness of findings [43] and to identify discrepancies and disagreements. All interviews were audiotaped; transcribed verbatim, removing any participant identifiers; and coded using ATLAS.ti version 7, a qualitative analysis program manufactured by Scientific Software Development [44].

#### Dyadic Analysis of Interviews With Parents and Adolescents

As the original focus of this study did not include dyadic-level analyses, a direct comparison approach could not be used because of differences in questions asked of adolescents and their parents. However, during the analysis of parent and adolescent interviews, similar descriptions of events or ideas by parents and their children created an organic opportunity to examine dyadic-level themes by way of content analysis. Using the Eisikovits and Koren model of dyadic analysis [43], matched adolescent-parent dyadic transcripts were reviewed by horizontalization and cross-analysis for overlaps and contrasts between text, subtext, descriptive, and interpretive levels, with the description of an event that occurred in SM (eg, cyberbullying of the adolescent from the perspective of the adolescent and from the perspective of the parent) as the unit of analysis. Dyadic transcripts were reviewed by independent coders (the first and second authors) who noted these horizontal themes, and discrepancies were settled by team consensus.

#### Analysis of Individual Interviews With Parents

The data analytic approach to the description of parents’ experiences with their child’s SM use used the technique of qualitative description. As described by Sandelowski [45], qualitative description refers to a thematic content analysis where findings aimed to stay close to the data as opposed to being overly interpretative. Using a content analysis approach [46], the first 4 interviews were reviewed independently by 2 investigators using an initial codebook based on the interview script. An updated list of codes focusing on key areas of interest was generated, with additional review by a senior member of the research team. Subsequently, the rest of the interviews were coded by 1 investigator and then reviewed by the senior author who made additions and/or changes. Additions of new codes or changes in code definitions were determined via consensus among the research team. The final sample size was determined by content saturation, which refers to the point at which adequate information is gathered to meet the purposes and goals of the research [47].

## Results

### Participant Characteristics

Of those who completed the study (23 parents and 23 adolescents), the average age for adolescents was 16 years (range 13-20 years) and the average age for parents was 46 years (range 37-55 years). Most adolescents (18/23, 78%) and most parents (19/23, 83%) were female, and most adolescents (20/23, 87%) and parents (21/23, 91%) were White (3/23 adolescents, 13%, and 2/23 parents, 9%, were African American), which reflects the demographics of patients seeking care for depression at the 2 sites. At the time of enrollment, adolescents on average had received treatment for depression over 25.4 months (range 3-84 months). Most parents (19/23, 83%) and adolescents (22/23, 96%) used SM. Parents most frequently reported using Facebook (18/23, 78%), whereas adolescents’ SM use was more diverse across platforms (10/23, 43%, used Facebook and 4/23, 17%, used Twitter, Tumblr, and Instagram).

### Dyadic Analysis of Parent and Adolescent Interviews

We first summarized themes based on dyadic analysis of the interviews, on which we directly compared child and parent interpretations of shared events. This analysis aimed to understand parents’ and adolescents’ mutual and divergent interpretations of adolescents’ use of SM in their daily lives. These themes included SM as a form of expression, the function of SM, SM as a space to discuss depression with others, interacting with strangers on SM, and parental monitoring of adolescents’ SM use.

#### SM as a Form of Expression

Parents and adolescents generally disagreed on SM being a medium for individual expression. Adolescents viewed SM as a forum for honest expression of themselves and their emotions, whether positive or negative. Adolescents elected to express themselves through song lyrics that capture their emotions or what adolescents described as ranting about an event that happened to them. Parents, however, saw much of what their adolescent was posting on SM as inconsequential or something to do when they felt bored and that it could interfere with other activities in the adolescent’s life. These themes are demonstrated in the following quotes from a parent and child dyad who had divergent views on adolescents’ SM expression:

Just to post like whatever you want. Because it’s your Twitter, so you can just say how you feel, like what you’re doing that day. Just anything in general really. Like anything that you want. So, it shouldn’t matter like what you post.

Child perspective; age 16 years

But when I saw what she—all the song lyrics she was putting on there, that caused me to have concern and... I just brought it up. And she thought it was funny. And she’s, and you know—I said, ‘I don’t understand why you want to put these lyrics out there,’ because they weren’t good lyrics. And she says, ‘Well, I’m expressing myself.’ And I say, ‘But why do you have to tell everybody? Like why’—I don’t get it. I just don’t get the whole thing. I’m just old-fashioned.

Parent perspective

#### Positive and Negative Functions of SM

Some parents and adolescents mutually described the potential of SM to be a protective, healing, or supportive space or a place to seek positive content. In other cases, parents and adolescents both offered clear examples of and reflections on the harms of SM use. For example, one parent was concerned about posts or forums that encourage self-harm behaviors (eg, cutting), which aligns with her daughter’s concerns—who had a history of intentional self-harm—and was worried that such forums or posts would encourage her daughter to cut herself. Other examples had greater discrepancy, which typically occurred when one member of the pair was more ambivalent and the other had a stronger point of view. The following quotes present discrepant perspectives between a parent and child dyad on the use and utility of SM:

So I think that if I do something on that website, like if I make an edit to just something that I enjoy and I’m interested in, that’s something that’s good, and I can read the feedback that I get from it. And so as long as I’m busy rather than if I look up the tag of #sad, that might make me more sad. But it’s kind of like, I get some sort of satisfaction from looking at sad pictures because it’s something I can relate to, which is kind of weird.

Child perspective, age 14 years

She spends an inordinate amount of time. And while the people on there, cool, some of them have the same experiences and stuff, she’s also not getting the face-to-face social stuff that I think she really needs, especially somebody going through what she’s going through as far as the depression.

Parent perspective

#### SM as a Problematic Space to Discuss Depression With Others

Some parents and adolescents agreed that it was safer, more private, and more authentic to talk about depression with trusted friends offline; however, their reasoning was not always the same. Parents were concerned about negative consequences and expressed distrust with others on SM, or they preferred that their child talk to them directly. Adolescents, on the other hand, expressed worry about what others would think—that posting on SM would draw unwanted attention, be burdening to others, or simply felt it was not others’ business. These viewpoints are demonstrated by the following parent and child dyad:

I don’t want everybody to know my business, like what I’m dealing with on like a really personal level...a family member might see it and, well, they might tell another family member about it and it would just go around the whole family.

Child perspective; age 17 years

My thought’s that if they feel that way, he should come to his mother and father and talk to them about it instead of putting it out there for everybody else. Some people is cruel, and they’ll end up saying something that’ll make him worse.

Parent perspective

#### Risks and Benefits of Anonymous Interactions on SM

Parents and adolescents had differing opinions about talking with strangers or interacting anonymously on SM. Parents expressed concern over their child being friends or having followers that were strangers or the lack of privacy in SM. In contrast, adolescents perceived that having strangers as friends on SM was not problematic and normative. These divergent perspectives are described by the following parent and child dyad:

I think it’s because I was just younger and I didn’t care who I was friends with, or you know, like you want to have so many friends on there so you just add whoever, type of thing.

Child perspective; age 17 years

How dangerous it is, and, you know, some of these people are older, 20s, 30s, they ’re men. How she doesn’t need to be, they don’t need to see her pictures, they don’t need to know anything about her, they don’t know her period, that people are dangerous, you can’t trust any, you know, everybody. You know, that people can find out anything they want to know.

Parent perspective

#### Parents’ Monitoring of Adolescents’ SM Use

Parent and adolescent perspectives on monitoring were predominantly divergent. In some cases, parents’ attempts to view their child’s SM content to prevent negative experiences left adolescents feeling resentful, particularly when the adolescent was excluded from this process (eg, a parent looking though their child’s phone without the adolescent’s permission). Furthermore, when parents restricted SM use, some adolescents defied their parents’ wishes by creating secret accounts on SM or by engaging with strangers. In other cases, parents considered monitoring strategies that would protect the parent-child relationship, which might include having honest conversations with their child about privacy or safety issues surrounding sharing of information on SM. These efforts corresponded with adolescents citing examples of choosing not to disclose private matters on SM; however, adolescents rarely contributed their decisions regarding privacy to their parents’ monitoring efforts, as is exemplified in the following quotes from a parent and adolescent dyad:

I really do think that she’s too smart to share it with strangers. I mean, I really think I’ve educated her in that, you know, strangers are never your friends, and they’re not who they say are—I think she’s wiser than her years when it comes to that, but I’m sure a lot of parents think that and then something tragic happens.

Parent perspective

If it’s something private, I think—I mean just from my opinion, I think, I just keep it to myself...I think that people would see it and just kind of like, maybe feel bad or like—and I just feel that they would know my personal business. And it’s just like not necessary.

Child perspective

### Analysis of Individual Parent Interviews

Drawing from the frequent divergent interpretation of adolescents’ SM use and behaviors, themes were explored to identify the ways in which parents of youth with depression within this sample gain knowledge of their child’s SM use and parents’ attitudes and experiences with SM monitoring.

#### Parental Expectations

Parents had a variety of expectations both toward their adolescents’ capacity to use SM in a healthy manner and in the potential of monitoring to have an impact on their child’s SM use. Parents generally expected their child to use SM in a way that was healthy. As described in the following quote, some parents felt that their expectations were met, describing a sense of trust in their child to create and maintain prosocial SM relationships and to express their opinions and experiences of depression in a way that is healing:

I trust her, you know, quite a bit, really—I mean, fully—with the use of it. I think she’s got a good head on her shoulders, and, so, yeah, I think she’s managing her own positive experience of it.

Alternatively, other parents’ expectations were unmet. They also had concerns that their adolescent was engaging in maladaptive behaviors such as oversharing (or being too trusting of sharing personal information on SM), which parents believed may have negative consequences, such as bullying, embarrassment, or future impact, on employment opportunities. Some informants were surprised to find their child’s peers discussing risky behavior on SM (eg, sharing sexually explicit content). One parent offered an example of discomfort with photos shared by her child’s friends:

...my daughters have a lot of friends with inappropriate photographs, and I don’t think that they think they’re inappropriate, but...I don’t think teenage girls should be in bathing suits on the internet.

In addition, parents expressed both positive and negative expectations that their monitoring efforts would actually have an impact on their adolescents’ engagement with SM. Some felt capable of intervening to improve their adolescent’s SM interactions, for example, having an open conversation about cyberbullying. Alternatively, others were concerned that SM was too difficult to regulate or control and that intervening would be ineffective. The following quote offers an example of a parent’s negative expectation:

...it’s not a battle I choose to fight. I’m not going to—you can’t—the internet in general is basically an unlimited freedom. You can see and do just about whatever your imagination can come up with.

#### Adolescent’s Disclosure to Their Parents

Descriptions or perceptions that adolescents are telling their parents about their SM use or the impact of SM use on their mood varied. In some cases, when adolescents accepted their parent’s monitoring, they were willing to openly discuss their SM use and its impact on them with their parents. One parent offered an example of her child openly disclosed information about her SM use:

...she’s pretty—[name] is I would say much more open with me than, than I, than certainly I ever was with my parents...I just felt like my mom was so far, you know, like way into my business in the first place, that I didn’t share, but [name] and I have a, have a pretty good relationship, I think and like she has no problem. Like she’ll come to me for advice and say, ‘What should I say to this person?’

Selective disclosure of behaviors was also observed, meaning that adolescents hid some aspects of their SM use from their parents in fear of a negative consequence. In these cases, adolescents feared that negative SM experiences may lead to parents deactivating their SM accounts, or they fear their parents will respond with rejection or disapproval.

#### Parental Knowledge

Several parents found it difficult to be knowledgeable about their children’s SM behavior. As described in the following quote, parents reported being unaware of which platforms their child used and did not know if their adolescent was bypassing their rules regarding SM use:

I asked her not to get on Facebook anymore. And then she snuck, and she got on Facebook a second time, using a different name.

Some parents acknowledged that their adolescents had hidden concerning SM interactions from them. For example, one parent noted that her daughter hid photos posted on SM of her self-injuring:

Before she had her attempt of suicide, she had a GifBoom account that was very dark, and we didn’t find out about it until later, but like had pictures of cutting—it almost was like a support group for that.

Other parents were more secure in their knowledge of their child’s SM interactions, and at times, this impacted adolescents’ SM behavior, often reducing personal content disclosed on SM. In the following quote, a parent described how their child being aware of familial monitoring corresponded with reduced sharing on SM:

Well, I have to be honest, she doesn’t really share too much online from a Facebook standpoint, regarding how she feels or her depression, because she has many family members who are her friends on Facebook. And I think, and I’m trying to get into her mind, and I’m thinking that she doesn’t put a lot of that out there because of who they are. Like if it’s her Nana, which is her grandmother, or her grandfather, or her aunts and uncles, she doesn’t put a lot of that out there, because she knows that they will read it.

#### Parental Solicitation

To gain knowledge of their child’s SM use, parents engaged in solicitation. In some cases, solicitation involved directly asking the child about their SM use. Parents engaged in this direct form of solicitation both in person and on SM, predominantly in instances when they felt their child was having negative experiences on SM or based on a belief that SM was inherently harmful (eg, contributing to their depression or encouraging risky behavior). Parents not using this direct form of solicitation described not wanting to *hover* or be controlling and preferred trusting in their child to maintain healthy SM behavior.

Parents also engaged in indirect forms of solicitation, which may have included a parent looking through their child’s phone or knowing the password to their SM accounts. Some parents felt this was an important way to learn previously unknown information about their child, such as new disclosures of risk-taking behavior described on SM or even to have a window to how the child feels or experiences their depression in a way that adolescents may not communicate with their parents. In the following quote, a parent described her experience with learning the utility of SM in providing information on her child’s emotional state:

Her father and I both were very against her sharing these things online, or really even, we kind of wanted her to just not even have an online account or anything. But then we kind of were, I don’t know, awakened to the fact that this is kind of the only window you have into your kid’s emotions sometimes. You know, teenagers especially don’t like to tell their parents what’s going on or to talk about how they’re feeling. And sometimes that’s how you see how they’re really feeling, is by some of the stuff they’re posting, even if they don’t realize that they’re showing it, you can kind of just see their different moods. I think it definitely has given us some, I don’t know, opportunities to talk that maybe we wouldn’t have had before. Especially—it’s very easy for kids to just come home and say, ‘Yep, school was fine. I’m fine.’ And the that’s it, you know.

Adolescents were more accepting of indirect forms of monitoring when there was an agreement between the parent and child of how this would be done. For example, one adolescent noted that their parent having their account password information led to openness in communication surrounding SM. To protect their child’s privacy and the parent-child relationship, some parents used other sources such as asking a friend, sibling, or other relative to assist in monitoring or using monitoring software.

#### Parental Control

Efforts to control adolescents’ behaviors included restricting sites perceived to be harmful and attempting to restrict certain SM behavior. One parent described how their efforts to restrict their child’s SM use were brought about by a desire to protect their child from harmful SM content:

Honestly right now, at this point in time, I have blocked most of the social networking sites from my daughter because of some of the things that I have seen on her wall and some of the things that I have seen that she posted. Because she’s going through this, let’s call it a difficult time in her life right now, I don’t think that she needs to rehash and read, and see all these different things because it’s just open— everything that is imaginable is on these social networking, and I just, I’m—I guess I’m trying to, to the best of my ability, is to protect her from seeing these different things...

Furthermore, some parents limited their adolescent’s SM use after their child did something of which they disapproved. Disciplinary actions included restricting access to a certain SM site entirely or taking the child’s phone away. In the following quote, a parent related her experience with restricting her child’s access to Facebook:

So, I asked her not to get on Facebook anymore. And then she snuck and she got on Facebook a second time, using a different name. And the same thing was happening...so then finally after she did it the third time, we told her absolutely, positively not. She wasn’t allowed to go even into Facebook to read it, to read anybody’s comments, to even talk to anybody. She’s not, at this point she’s not allowed on Facebook at all.

Rule setting was enforced through both direct and indirect means of solicitation, including the use of monitoring software or by asking designated members of the child’s social network (eg, a friend of the parent who is monitoring the adolescent on behalf of the parent). Attempts to restrict SM behavior varied by age, where some parents restricted the use of certain SM sites or any site before a certain age. Parents who did not engage in restriction reported a sense of powerlessness or loss of control. One parent described how she wished she could set rules but felt doing so would be ineffective and could damage her relationship with their child:

I wish I had the nerve to tell her she wasn’t allowed to use it but it would just be such a barrier and it would just make her hate me and not talk to me, and there’s no point—like I said, like I prepare her for the adult world, in a year and a half she’ll be at college, and I don’t want her to go off the deep end then and say, like, ‘My mother would never let me do this, so now I’ll show her...’

Another parent described a perception that attempts to control are ineffective and correspondingly a sense of lack of control:

We tried at first to completely like eliminate her having them, but we found that when you do that they just kind of find ways to do it behind your back, and then you have no way to know what’s going on. So, she voluntarily will show me a lot of this stuff. You know, I still do fear that there’s things out there that I’m not seeing. But I don’t know if there’s ever a way to monitor 100%. You’re almost kind of kidding yourself if you think you are.

## Discussion

### Principal Findings

This study uses rich qualitative inquiry to explore child and parent perspectives toward SM use and monitoring for adolescents with depression. Our study describes the experiences of adolescents with depression using SM to seek autonomy from their parents and the parents attempt to maintain knowledge of their child’s SM use to offer protection because of concerns of a potentially unsafe environment for their vulnerable child. Although previous research has described the struggles between adolescents’ and parents’ overuse of SM [22], this study’s dyadic analysis of the perspective of adolescents with depression and their respective parents is unique. SM presents challenges through risky SM behaviors, negative SM experiences such as cyberbullying, and worsening mood [11,39,48,49], to which adolescents seeking clinical treatment for depression are especially vulnerable. SM also presents opportunities for social connection and supportive interactions, which are especially important for youth facing depression [50]. Conflict over the use of SM in the context of depression may create added challenges that youth without depression and their parents or caregivers may not have to address. This study offers findings that have implications for clinicians engaged in the treatment of adolescents with depression, who are heavy users of SM. Especially in the current context of the COVID-19 pandemic, clinical guidance for parental monitoring of SM is critical.

The findings from the dyadic interviews showed that although a few parents and adolescents agreed about the use and function of SM, most had discrepant perspectives. Discrepancy often stemmed from a difference in value placed on autonomous SM expression versus protection from the risks of SM use. These views can be considered with a report released from the United Nations International Children’s Emergency Fund [51], which considers children’s rights for digital privacy. In accordance with the United Nations Convention on the Rights of the Child [52], this report suggests that children have a right to digital privacy and that parents have a dual responsibility—both to protect their adolescent from web-based threats and to encourage free expression by their adolescent in web-based spaces. This dual responsibility appears to create conflict and discrepant views between adolescents and their parents.

For parents attempting to balance this dual responsibility, United Nations International Children's Emergency Fund’s report suggests the extent to which privacy and access to SM is given should be considered based on the child’s evolving capacity, for example, their age and maturity level. In the case of adolescents with depression, a child’s capacity may also include the potential for heightened vulnerability to harmful SM experiences. At the same time, adolescents with depression report finding value in having a venue to express themselves emotionally and receive support through trusted SM friends. Furthermore, some studies found that these protective interactions had a healing effect, which positively influenced their mood. This suggests that although parents should take measures to protect their child from SM-related risks to their safety, they should also look for opportunities to allow their children to engage freely, particularly with supportive peers on SM.

The results of the individual interviews can be placed within the context of conceptualization of parental knowledge by Kerr and Stattin [23]. The sources of parental knowledge they have identified—*adolescent disclosure, parental solicitation, and parental control*—appear to be salient in understanding how parents engage in surveillance and monitoring of SM behavior of adolescents with depression. Consistent with the literature [25,26], adolescents voluntarily disclosed information about their SM use to their parents when they accepted their parents’ monitoring strategies but were less likely to disclose when they perceived the potential for a negative consequence. When teens felt comfortable, they were willing to discuss their SM use and its impact on their mental health with their parents. Due to concerns about their children engaging in risky SM behavior, several parents were keenly interested in gaining information either by directly asking their child about their SM use or through overt or covert methods of viewing their child’s actual SM content. Parents’ engagement in covert forms of solicitation are potentially concerning because privacy invasions have been associated with increased secrecy and lower parental knowledge [31,32]. Parents engaged in a variety of control techniques, that is, rule setting surrounding their child’s SM use and, in some cases, violation of parents’ rules resulting in disciplinary actions. When parents engaged in controlling behaviors without their child’s knowledge or buy-in, teens were not entirely honest with their parents about their SM experiences. When parents engaged in privacy invasions, adolescents became secretive and, in some cases, engaged in risky SM behavior without their parents’ knowledge.

These results suggest that there is a unique context for parents of adolescents with depression for engaging in SM monitoring and unique consequences for their child’s acceptance of their chosen monitoring strategies. The positive consequences of open parent-child communication that fosters voluntary disclosure are significant. Parents can be more aware of their child’s SM activity to protect them from the negative influences of SM while fostering positive influences. Likewise, the negative consequences of SM monitoring strategies that contribute to a lack of knowledge are also considerable. As was evident in these interviews, when adolescents with depression do not accept their parents’ use of SM monitoring, they have the potential to withhold information from their parents about risky SM communication, such as posting about self-harm or communicating with strangers. Lacking such information stifles parents’ capacity to protect their child from harm. Further, restriction techniques that limit access to supportive SM interactions may have the consequence of reducing the protective influences of SM.

### Implications for the Clinical Care of Adolescents With Depression

Understanding parent and child perspectives toward SM use and strategies parents deploy to learn about the SM activity of their child with depression could offer valuable insights toward clinical intervention planning. For example, the strategies parents use to procure information about their child’s SM use could either support or discourage the child’s voluntary disclosure of risky SM interactions or negative experiences. Such voluntary disclosure may be the most effective way for parents and clinicians to be knowledgeable about the level of risk within the child’s SM environment. In addition, adolescents’ voluntary disclosure to parents could result in a greater awareness of the protective aspects of their SM use, including sources of support that adolescents with depression may find critical to preventing their depressed mood from worsening. Understanding the context of adolescents and parents’ perspectives toward SM use and monitoring could aid clinicians in opening lines of discussion that validate both parties’ unique concerns to move toward the goal of an effective and acceptable monitoring approach.

In 2019, the American Association of Suicidology (AAS) [36] released recommendations for parents and providers engaging in SM monitoring of adolescents at risk for suicide. According to the recommendations by AAS, clinicians should engage in a risk assessment of both the helpful and harmful influences of adolescents’ engagement with SM and use this information to build a crisis intervention safety plan. It is considered a best practice for safety plans to be developed by clinicians in collaboration with parents. When engaging in risk assessment, clinicians should consider ways by which SM can offer meaningful opportunities for emotional expression and support for adolescents with depression and work with parents to consider how engaging in restrictive strategies could affect these helpful aspects of SM use. Clinicians should offer parents caution with strategies that involve privacy invasions that have the potential to weaken parent-child connections and may have the unintended consequence of placing children at higher risk. The risk assessment should also consider potential negative influences of SM, such as exposure to harmful or hateful content, and engage adolescents in considering ways to openly discuss negative SM experiences with their parents.

The Family Media Plan developed by the American Academy of Pediatrics [53] may be instrumental in developing a mutually agreeable plan for parents and adolescents. This plan offers an opportunity for open discussion about critical topics such as screen time, use at important times of day such as while doing homework or before bed, appropriateness of media content, and rules for digital safety. Clinicians could be trusted confidants to adolescents with depression and their parents when considering these topics and work together with the family to find a middle ground between parents’ desire for protection and adolescents’ desire for autonomy toward a mutually acceptable monitoring approach.

### Implications for the COVID-19 Pandemic

The unique context of the COVID-19 pandemic is especially relevant and important for the SM use and parental monitoring of adolescents with depression. Our data reflect the importance that adolescents with depression place on maintaining prosocial SM peer interactions. During the pandemic, when youth face challenges associated with the loss of face-to-face social interaction, maintaining connection with supportive peers on SM is important to mitigate feelings of social isolation, a significant risk factor for suicide [5,54]. At the same time, parents of youth with depression, who our data reflect are concerned about their child’s exposure to negative SM experiences, may be especially worried about heightened risks associated with adolescents’ increased SM activity. This context places a demand on parents to monitor their adolescents’ SM use; however, this comes into conflict with significant economic, social, and health challenges that affect families’ daily lives [55].

As adolescents and their parents face unprecedented challenges, clinicians of youth with depression have an important role in helping families find a balance between concerns for safety on SM and autonomous communication with peers. Given the risk of social deprivation that ongoing physical distancing measures may have on adolescents, clinicians should work with parents to consider risks of limiting their child’s access to supportive SM peers, while also offering guidance to reduce exposure to negative SM content (eg, cyberbullying or conversations or images pertaining to self-harm). Clinicians may benefit from using resources from Common Sense Media, which provide guidance for parents in safeguarding their children’s physical and mental wellness as they access digital media during the pandemic [56]. In addition, the Suicide Prevention Lifeline’s collection of web-based resources to support healthy coping during the COVID-19 outbreak may be beneficial contributions to safety plans for adolescents facing pandemic-related mental health challenges [57].

### Strengths and Limitations

A key methodological strength of this study was the conduct of separate interviews with parent and child dyads, which allowed for analysis at both the individual and dyad levels. First, the separate analysis of parent and child interviews allows each informant to tell the story from their own perspective [43]. As adolescents and their parents often have incongruent views of the same events [58], dyadic interviews provide an opportunity to highlight conflicting perspectives on SM use and monitoring. Second, the collection of separately conducted parent and child interviews helped capture the individual within the dyad, without sacrificing the dyadic perspective [43]. Finally, the ability to triangulate perspectives at both an individual level and dyad level increases the capacity to broaden knowledge and contextual understanding of a phenomenon [43].

This study offers a unique contribution to the literature by exploring parent and child perspectives toward SM use and monitoring of adolescents with depression. Despite the novelty of this study, it has limitations. First, this study was not intended to be a dyadic analysis of how both parents and their adolescents with depression perceived the adolescent’s use of SM. Some aspects of the parent-child relationship related to SM use and monitoring may be unknown because they were not shared during the interviews. Second, the sample is predominantly White and female and from an academic medical institution, where many of the patients visiting the clinic may have had more severe forms of depression and, therefore, may not be representative of adolescents with depression and their respective parents. Moreover, these same patients were brought to the clinic by their parents, suggesting that these parents may be more proactive in their children’s health, which may include their child’s SM use. Third, the same interviewers conducted these dyadic interviews. In this situation, although the interviewer assured confidentiality and interviewed participants separately, the other party was virtually present in the interview space. This may make the other party (whether parent or adolescent) be selective of what they will tell the interviewer [43]. Finally, we have framed the relevance of these findings within the COVID-19 pandemic that has observed a sharp increase in SM use alongside a diminishment of parents’ availability for monitoring [59]; however, these data were collected before the pandemic’s onset. Therefore, potential differences in adolescents’ and parents’ perspectives toward within the current context and the evolving nature of SM use may not be represented. Despite these limitations, this study provides helpful insights into the challenges adolescents with depression and their parents face in approaching safety and acceptable SM monitoring.

### Conclusions

Our interviews described the experiences of adolescents with depression and their respective parents on adolescents’ use of SM in their daily lives, a topic that bears particular relevance during the COVID-19 pandemic when SM use has risen dramatically in response to physical distancing measures. The findings highlight the conflict over adolescents’ growing need for autonomy versus the parents’ need to protect their child with depression because of concern for their child’s heightened vulnerability to the risks of SM use. Parents try to balance these 2 components by obtaining knowledge of their child’s SM use through their child’s voluntary disclosure, by soliciting information from their child, or through parental control techniques. Our study has highlighted the need for clinicians to partner with families to identify mutually agreeable monitoring strategies that meet both the parents’ desire to be knowledgeable about their vulnerable child’s SM use and the adolescents’ need for independence in their SM interactions.

## Abbreviations

AAS: American Association of Suicidology
SM: social media
